# Time from injury and age interact in relationship with perceived quality of life outcomes following vocation-focused neuropsychological rehabilitation

**DOI:** 10.3389/fpsyg.2023.1047615

**Published:** 2023-02-10

**Authors:** Ayala Bloch, Tal Shany-Ur, Limor Sharoni, Narkis Bar-Lev, Tali Salomon-Shushan, Sari Maril, Eran Druckman, Dan Hoofien

**Affiliations:** ^1^Department of Psychology, Ariel University, Ariel, Israel; ^2^The National Institute of Neuropsychological Rehabilitation, Tel Aviv, Israel; ^3^Department of Psychology, The Hebrew University of Jerusalem, Jerusalem, Israel; ^4^Druckman Research and Statistics Lab, Rishon Lezion, Israel; ^5^The School of Behavioral Sciences, The Academic College of Tel Aviv-Yaffo, Tel Aviv, Israel

**Keywords:** vocational rehabilitation, brain injury, time since injury, perceived quality of life, neuropsychology, employment

## Abstract

At the group level, community-based neuropsychological rehabilitation interventions with a vocational focus are generally effective among individuals with brain injuries. However, individual participants vary significantly in the extent of their improvement, prompting attempts to elucidate individual, injury-related, and environmental factors affecting prognosis. In this study, we examined the relationships between one such factor – “time from injury” (the time between injury and intervention) – and two outcome measures: employment status and perceived quality of life (PQoL), in 157 brain injury survivors, before and after a holistic neuropsychological vocational rehabilitation program. We also examined whether relationships between the variables were moderated by age at onset of treatment and injury severity. In the entire sample, both the proportion of employed participants and average PQoL increased following program participation. Neither, time from injury, severity, nor age at onset of treatment predicted the increase in employment proportion, and severity was not a significant predictor of PQoL. However, an interactive effect indicated that when treatment was started at a younger age, longer time from injury predicted higher levels of PQoL, but when treatment was started at older ages, longer time from injury predicted lower levels of PQoL. When interpreted alongside existing literature, these results suggest that delaying vocational components of rehabilitation can be beneficial for younger participants, while the effectiveness of vocational rehabilitation can be maximized by starting as early as possible among older participants. Most importantly, regardless of age, it appears that vocational rehabilitation can be effective even when initiated many years after injury.

## 1. Introduction

Acquired brain injury (ABI) caused by trauma or disease can affect physical, cognitive, and emotional functioning, as well as behavior (Koponen et al., 2002; Cattelani et al., 2010; Doering and Exner, 2011). These in turn determine post-injury reintegration into the community and particularly the ability to enter or rejoin the workforce (Grauwmeijer et al., 2012; Howe et al., 2018).

Although some degree of neural damage caused by ABI has generally been considered irreversible (Kochanek et al., 2007), there is a large body of research demonstrating the capacity for neural recovery and reorganization following injury (Nagappan et al., 2020). Accordingly, substantial improvements in functioning can be achieved through rehabilitation processes following ABI (Maas et al., 2022). Large-scale longitudinal studies conducted through clinical care and research networks in the United States (Dijkers et al., 2018) and Australia (Ponsford et al., 2021) have both shown that early and continuous rehabilitation can reduce the length of stay in hospital and mitigate socioeconomic burdens. Unlike early rehabilitation efforts in the acute stage, which tend to emphasize physical healing (Oberholzer and Müri, 2019), neuropsychological rehabilitation during the post-acute and chronic stages focuses on improving or learning to cope with cognitive, mental, and behavioral deficits (Teasell et al., 2007; Wilson, 2008). The success of long-term neuropsychological rehabilitation can be expressed in diverse measures of functioning, such as greater independence in daily activities, increased mobility, broader social integration, and return to previous or new employment (Seagly et al., 2018).

Many community-based holistic neuropsychological rehabilitation programs focus, among other things, on vocational rehabilitation for individuals who have experienced brain injuries (Grandisson et al., 2016). In accordance with the holistic bio-psycho-social model of rehabilitation (Cope, 1995; Tate and Pledger, 2003; Leonardi and Martinuzzi, 2009), holistic vocational rehabilitation programs are thus named because they address the participant as a whole. These programs, which define reintegration into employment as a primary goal, therefore encompass various interventional components, which can include psychotherapy and cognitive training as well as vocational assessment, counseling, training, and job placement (Malec and Degiorgio, 2002; Malec and Moessner, 2006; Cullen et al., 2007). The emphasis on return to work in post-acute rehabilitation is rooted in research indicating decreased employment rates among survivors of ABI (Andelic et al., 2009; Forslund et al., 2017) alongside clear associations between employment and better physical, social and psychological well-being (Ponsford et al., 2008; Andelic et al., 2009).

Several studies have addressed the efficacy of community-based neuropsychological rehabilitation interventions with a vocational focus (Diller and Ben-Yishay, 2020; Domensino et al., 2021). Overall, such programs appear to be beneficial, not only with respect to general functioning and employment status but also in terms of subjective life satisfaction (Shany-Ur et al., 2020). Alongside the more objective measures associated with core program goals, evaluating satisfaction with life as an outcome of rehabilitation is in line with current perspectives that place the person, and personal aims, at the center of rehabilitation interventions (Bridger et al., 2022).

While studies generally show that vocational rehabilitation program outcomes are effective at the group level, individual participants vary significantly in the extent of their improvement (Allanson et al., 2017; Libeson et al., 2022). To assess an individual’s suitability for vocational rehabilitation in terms of anticipated prognosis for improvement, rehabilitation professionals must consider a complex combination of factors. Some of these are external, such as the number of hours of intervention provided by a program, or the extent of support provided by family members.

Other factors potentially affecting rehabilitation outcomes relate to the individual and the injury. Some studies have shown that age affects prognosis following rehabilitation (Brown et al., 2005; Sigurdardottir et al., 2018). Other work shows that level of depression at the onset of rehabilitation following brain injury is negatively correlated with successful outcome (Coetzer et al., 2011). Severity of injury, as measured by the Glasgow Coma Scale, reported post-traumatic amnesia, or length of stay in intensive care, has also been associated with differences in post-rehabilitation functioning (e.g., Dikmen et al., 2010; Sigurdardottir et al., 2018), with greater severity related to poorer outcomes.

Another key factor considered in assessing prognosis for community-based vocational rehabilitation is “time from injury,” or the amount of time that has passed between the brain injury and the onset of intervention (Powell, 2002; Andelic et al., 2012; Aas et al., 2018; Königs et al., 2018). Like the other variables noted above, time from injury has been of interest to both clinicians and health policy makers in determining the optimal time to introduce vocational interventions during the broader long-term community-based rehabilitation process (Lannin et al., 2021).

As noted above, vocational rehabilitation generally becomes an option when the more physically-focused acute rehabilitation process has been deemed sufficient. Reaching a level of physical healing that makes it possible to perform the tasks required by vocational rehabilitation designates the earliest possible start point.

Beyond this, however, research attempting to determine whether there is a “window of opportunity,” during which vocational rehabilitation is more likely to succeed, has produced varied results. This body of work has addressed two primary questions: (1) when is the optimal time to start rehabilitation, and (2) can rehabilitation be valuable regardless of time from injury, or is there a point when it becomes “too late” to start.

Some studies have shown that functional outcomes including return to work are better when multi-disciplinary rehabilitation is started earlier following moderate to severe brain injury (Andelic et al., 2012; Königs et al., 2018). Other work, however, showed that time from injury did not predict vocational rehabilitation outcomes at all (Aas et al., 2018). This raises the possibility that other variables, including personal and injury-related characteristics, might moderate the relationship between time from injury and rehabilitation outcome. The result would be that the optimal window for vocational rehabilitation varies between individuals, precluding a clear association between these two variables at the group level.

In the current study, we examined the relationship between time from injury and two outcome measures: employment status and perceived quality of life (PQoL), following a holistic neuropsychological vocational rehabilitation program. We also examined whether these relationships were moderated by age at onset of treatment and severity of injury as measured by length of coma (LOC). Both of these variables are presumably related to clinical and behavioral determinants of rehabilitation outcome, such as self-awareness, adherence, and maturity in the case of age, and cognitive abilities in the case of severity. Essentially, we aimed to determine if starting rehabilitation earlier or later would be associated with better outcomes, and whether the pattern displayed would be consistent throughout the group or vary based on age and severity of injury. Given the clear role of employment status in improving post-ABI outcomes at the individual, familial, and societal levels (Andelic et al., 2009), we conducted the current research to extend previous findings and further elucidate key contributors to successful vocational rehabilitation.

## 2. Method

### 2.1. Participants

Participants were 157 (48 female, 109 male) individuals who had experienced ABI (see Table 1 for nature of injuries) and received treatment at a community-based holistic neuropsychological rehabilitation center. Age in the entire sample ranged from 19 to 59 years (*M* = 34.90, SD = 10.65). As all participants were first assessed at Time 1, upon beginning treatment, their age corresponds with the age of onset of treatment measure. Education ranged from 7 to 21 years (*M* = 12.92, SD = 2.14). Length of coma (LOC) served as a measure of severity. Of the entire sample, 82 participants (52.2%) experienced comas while 75 (47.8%) did not. All participants were considered “working age” (i.e., not retired). About 13% were employed at T1 but they, like all program participants, were actively attempting to find or change jobs; vocational reintegration is a specialization of the rehabilitation center and a primary goal in all the programs it offers. Current employment status and details of employment history were assessed and validated by program staff.

**Table 1:** Descriptive statistics for the study variables.

	Number	%	*M*	SD	Range
Age			34.90	10.65	19.00–59.00
Gender					
Female	48	30.6			
Male	109	69.4			
Nature of injury					
TBI	93	59.2			
Non-TBI	64	40.8			
Length of coma (days)			0.52	0.50	0–9.00
Time from injury (years)			3.08	1.94	0.50–9.00
Age at onset of treatment			34.90	10.65	19.00–59.00
Employed					
Time 1	20	12.9			
Time 2	74	47.7			
Perceived quality of life					
Time 1			5.42	2.01	0.50–9.85
Time 2			5.74	2.03	0.00–9.60

Information on the employment status of two participants was missing (*n* = 155). TBI, traumatic brain injury – including traffic accidents, work accidents, falls, acts of violence; non-TBI, non-traumatic brain injury – including stroke, brain tumor, epilepsy, hypoxia, other non-degenerative medical conditions affecting the brain.

### 2.2. Measures

#### 2.2.1. Perceived quality of life

The PQoL questionnaire (Patrick et al., 2000) addresses degree of satisfaction with various aspects of life. It comprises 20 items pertaining to physical health, self-care ability, social interactions, and functioning in various domains. In each item, participants are asked to rate their satisfaction in a particular area on a 0–10 scale, with higher scores indicating greater satisfaction. The questionnaire includes items addressing whether participants do or do not participate in various life activities. Item 20 is the only item asking directly about happiness, with the scale running from 0 “extremely unhappy” to 10 “very happy.” The average for all items serves as an overall score (maximal score = 10). The Hebrew version yielded high inter-item reliability (Cronbach’s alpha = 0.930, split-half = 0.904).

#### 2.2.2. Employment status

The employment status questionnaire comprises two items: “Do you have a paying job?” and “Are you a student at an institution of higher learning?” A response of “yes” to either one or both of these questions was considered “positive” and a response of “no” to both of them was considered “negative” on the dichotomous employment status measure.

### 2.3. Procedure

For this study, retrospective data was drawn from the files of relevant patients who received treatment at a community-based holistic neuropsychological rehabilitation center between the years 2005 and 2017. The database included information regarding the type of injury, severity of injury (length of coma), the time passed between the date of the injury and date the participant began rehabilitation at the center, and the results of the two outcome measures at two time points: before (T1) and immediately after (T2) the rehabilitation program.

All participants had taken part in one of two programs aimed at improving their functional outcome: comprehensive–holistic neuropsychological rehabilitation (CNR) and vocation-focused neuropsychological rehabilitation (VNR). Both programs contained at least three components: neuropsychological psychotherapy, cognitive rehabilitation, and occupational counseling and placement, but varied in scope and intensity in accordance with the needs and abilities of participants, as indicated by a broad neuropsychological assessment and the recommendations of the clinical staff. Brief descriptions of the programs are provided below. They have been described in detail in a previous publication (Shany-Ur et al., 2020, p. 134). Data for the current study were collected retrospectively and did not affect allocation of participants to the different programs; allocation was based on professional clinical considerations alone.

As an integral part of the pretreatment intake process, patients were asked if, in addition to participating in the treatment program to which they were allocated, they would be willing to participate in an ongoing, long-term follow-up study. They were informed that the primary aim was assessment of the rehabilitation institute’s treatment programs, as a basis for continual improvement of care. They were also informed that they would need to complete study measures at multiple time-points, including after program completion. All participants agreed to these conditions and gave informed consent. The study was approved by the institutional ethics committee.

### 2.4. Rehabilitation programs and placement

Senior staff members, all certified rehabilitation psychologists and clinical neuropsychologists, determined which patients were admitted to each program based on clinical considerations, to maximize fit between program characteristics and patient capabilities, disabilities, challenges, needs, goals, and prognosis. All interventions in both programs were supervised by certified rehabilitation psychologists and clinical neuropsychologists. Some were administered by psychology residents.

Comprehensive–holistic neuropsychological rehabilitation entailed 10 months of intensive treatments, 5 to 7 hours daily, 4 days a week. Groups of 5–10 participants attended a structured, obligatory curriculum of group (about 20 weekly hours) and individual therapy (about 4 weekly hours) sessions. Cognitive interventions addressed attention, memory, communication, executive functioning, and psycho-education about the brain and brain injury. Additional tailored interventions addressed functional skills, such as arithmetic, reading comprehension, and basic computer use. Psychological interventions included individual psychotherapy, group therapy, and vocational counseling.

Vocation-focused neuropsychological rehabilitation was also an intensive group-based day program. Patients spent two-thirds of their time in prevocational workshops resembling work environments and a third of their time in individual and group treatments. Groups were ongoing, with individual participants joining and leaving in accordance with their specific needs. Program length ranged from 4 to 18 months, with intensity increasing gradually from 2 to 5 days a week. Workshops included technical assembly, clerical and office tasks, carpentry, or gardening. Individual treatments included psychotherapy, cognitive rehabilitation, vocational counseling, and case management. Group therapy sessions included vocational, cognitive, psycho-education, and support groups.

### 2.5. Data analysis

All statistical analyses were conducted using IBM SPSS statistics version 25 and Process macro V4.0. The independent variables were age (in years) at onset of rehabilitation, severity of injury (LOC), and time (in years) between the injury and onset of rehabilitation. The dependent variables were PQoL and employment status before and after participation in a rehabilitation program. We examined gender/sex-based differences in the dependent variables and in the change in dependent variables from T1 to T2. As none were significant, we did not include gender/sex in further analyses. Change in outcomes following participation in the program were examined using a *t*-test (for PQoL) and a McNemar test (for proportion of employed participants). Pearson correlations between the study variables were calculated (see Ferguson, 2009 for a discussion of effect size interpretations). Correlations between length of coma, time from injury, age at onset of treatment, employment at Time 2, and PQoL at Time 2 were examined while controlling employment and quality of life at Time 1 (respectively), using partial correlations. This method enables examination of associations with outcome variables at Time 2 above and beyond Time 1, with significant correlations serving as a basis for the variables included in subsequent regression analyses. Finally, we conducted logistic regression (for predicting employment) and linear regression (for predicting PQoL) analyses, using Model 1 in the Process macro. To examine the interaction effect we found, we used the Johnson-Neyman technique, which examines simple effects along different values of the moderating variable. We used this method to examine the age at which, according to the sample data, there was a change in the significance of the effect.

## 3. Results

### 3.1. Participant characteristics

Descriptive statistics for the experimental variables (length of coma, time from injury, age at onset of treatment, employment status, and PQoL) are presented in Table 1.

### 3.2. Outcomes following program participation

As seen in Table 1, 72 participants (47.7%) were employed after participating in the programs, compared to 20 participants (12.9%) before the program. A McNemar test showed that this increase in the proportion of employed participants was significant (*p* < 0.001). Likewise, a paired-samples *t*-test revealed a significant increase in PQoL, *t*(156) = 2.32, *p* = 0.011, *d* = 0.19, between Time 1 and Time 2.

### 3.3. Correlations between study variables

Table 2 shows correlations between the study variables. Note again that we calculated correlations with employment at Time 2 and PQoL at Time 2 while controlling for these variables as measured at Time 1. We found no significant associations between LOC and each of the study variables. Time from the injury was negatively associated with age of onset of treatment. Time from injury and age at onset of treatment were not associated with employment or PQoL after participation in the program. Finally, employment after the program was positively associated with PQoL.

**Table 2:** Correlations between study variables.

	1	2	3	4	5
LOC	–				
Time from injury	−0.10	–			
Age at onset of treatment	−0.10	−0.18*	–		
Employed (Time 2)	−0.03	0.05	−0.08	–	
Perceived quality of life (Time 2)	−0.06	0.09	−0.07	0.28**	–

*p < 0.05, **p < 0.01. Correlations with employment at Time 2 and perceived quality of life at Time 2 were calculated while controlling these variables at Time 1.

### 3.4. Regression models for predicting employment and perceived quality of life

Table 3 shows the results of the regression analyses for predicting employment and PQoL. The logistic regression for predicting employment at Time 2 was not significant. The linear regression for predicting PQoL at Time 2 was significant, showing that LOC, time from injury, and age at onset of treatment explained 44.0% of the variance in PQoL. PQoL at Time 1 positively predicted PQoL at Time 2. In addition, significant main effects of time from injury as well as age at onset of treatment were found, alongside an interaction between these two variables.

**Table 3:** Regression models for predicting employment and perceived quality of life (PQoL) after rehabilitation.

	Logistic regression for predicting employment (Time 2)			Linear regression for predicting PQoL (Time 2)		
	*B*	SE	*p*	*B*	SE	*p*
Employed / PQoL (Time 1)	0.87	0.54	0.107	0.62	0.06	**<0.001**
Length of coma (LOC)	−0.16	0.34	0.636	−0.25	0.25	0.314
Time from injury	0.12	0.30	0.698	0.67	0.23	**0.004**
Age at onset of treatment	−0.01	0.03	0.782	0.05	0.02	**0.044**
Time from injury × Age at onset of treatment	0.00	0.01	0.791	−0.02	0.01	**0.005**
Model summary						
$\chi^{2}$ */F*		5.35			23.73	
*df*		5			5,151	
Pseudo *R^2/^R^2^*		0.046			0.440	
*p*		0.375			**<0.001**	

Significant *p* values are marked in bold.

**Figure 1:**
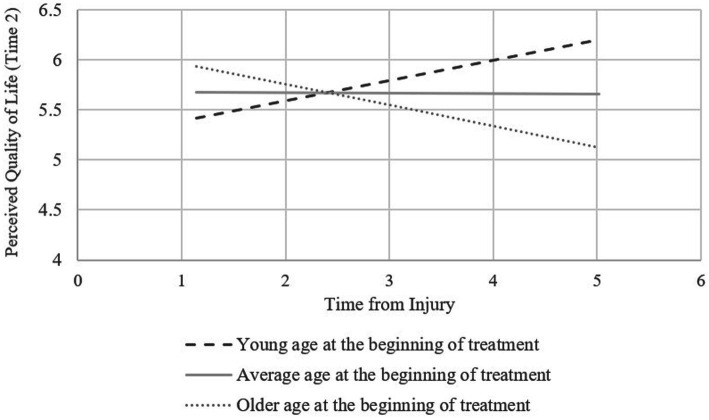
Simple effects of time from injury on perceived quality of life.

The results of the Johnson-Neyman analysis used to examine the interaction effect on PQoL indicated a significant positive effect for younger participants (under 27.37 years) and a significant negative effect for older participants (over 47.67 years). Figure 1 shows the simple effects of time from injury on PQoL for participants who started treatment at an age 1 or more SDs below the average, participants who started treatment at an age between 1 SD below and 1 SD above the average age, and participants who started treatment at an age 1 or more SDs above the average.

## 4. Discussion

The current results indicate that both the proportion of employed participants and PQoL increased following participation in the programs. Neither time from injury, severity, or age at onset of treatment predicted the increase in employment proportion. With respect to PQoL, however, while severity was not a significant predictor, there was an interactive effect of time from injury and age. Specifically, when treatment was started at a younger age, longer time from injury predicted higher levels of PQoL. Conversely, when treatment was started at older ages, longer time from injury predicted lower levels of PQoL.

Before discussing the effects related to time from injury, we note that the current results are in line with previous work indicating the general efficacy of holistic, community-based neuropsychological rehabilitation programs focused on return to work and improved well-being (e.g., Shany-Ur et al., 2020). The current study, like many others attempting to assess neuropsychological outcomes without compromising the ethical allocation of therapy, has methodological limitations that stem from the lack of a non-intervention control group. Still, the results show that participants spent an average of over 3 years at home following their injuries before beginning the program, unable to find suitable employment. This provides a basis for comparison, reinforcing the significant increase shown in the proportion of employed participants between Time 1 and Time 2.

We can therefore say that employment status, which represents the key aim of vocational rehabilitation, improved regardless of time from injury or age at onset of intervention. The significance of this finding is that vocational rehabilitation programs can help participants of different ages return to the workforce even after many years outside it, refuting the idea that there is a “deadline” for beginning intervention. This viewpoint might seem to be supported by studies indicating that employment rates among brain injury survivors tend to stay stable over time or even to decrease (Howe et al., 2018). However, the results of the current study suggest that the appropriate intervention, namely holistic, community-based neuropsychological rehabilitation with a vocational focus, can enable brain injury survivors to return to the workforce regardless of the amount of time they spent unemployed following their injuries.

Another key aim of neuropsychological rehabilitation, both vocational and otherwise, is improving perceived quality of life (Andelic et al., 2018). In the current study, we found that when participants were younger at intervention onset, greater time from injury was associated with greater rise in PQoL. In contrast, and in line with some previous work (Andelic et al., 2012; Königs et al., 2018), the older participants appeared to show greater increases in PQoL when they started rehabilitation earlier rather than later. We know that changes in physiological, cognitive, emotional, and behavioral processes continue to occur during the years following brain injury (Vasquez et al., 2018), both spontaneously and in response to intervention. The questions that arise, then, involve how younger and older adults might differ in the processes that they undergo during the time between injury and beginning rehabilitation, and the mechanisms that make this waiting period conducive to rehabilitation among the former and detrimental to the latter. Such mechanisms could involve, for example, motivation, vocational goals, environmental expectations, and neurophysiological and neurocognitive recovery, all of which are influenced by age (e.g., Senathi-Raja et al., 2010; Inceoglu et al., 2012). The findings of the current study are not sufficient to draw conclusions regarding such mechanisms. However, as detailed below, they provide justification and direction for extended research examining the interplay of other variables with time from injury in relation to vocational rehabilitation outcomes in younger versus older adults. Furthermore, the significant improvement in PQoL shown by the entire sample indicates that despite the advantage for older participants who began intervention sooner after injury, it was beneficial even when started later.

Unexpectedly, severity of injury did not affect the relationships between time from injury and either of the outcome variables, employment status and PQoL. Several studies have reported associations between injury severity and reintegration into employment (Swan et al., 2018). This, alongside the known relationship between severity and cognitive functioning (Gorgoraptis et al., 2019), suggested that severity might mediate the relationship between time from injury and rehabilitation outcome. This was not, however, the case. Severity did not interact with time from injury in predicting employment status or PQoL. It is possible that LOC, the specific measure of severity used in the current study and the only one available for its participants, was not sensitive or accurate enough to elucidate these relationships. Though frequently used and generally accepted (Walker et al., 2020), medical measures that, like LOC, are based on physiology immediately following injury might not accurately reflect its cognitive and behavioral implications over time. Indeed, recent work suggests that other measures of severity could be preferable (Tenovuo et al., 2021). Beyond this, it is notable that in the current study, participants were allocated to specific rehabilitation interventions based on a range of characteristics, some presumably associated with the severity of injury and its long-term implications. It is possible that this selection process offset the influence of severity on the relationship between time from injury and rehabilitation outcome to the extent that this influence could not be detected.

### 4.1. Limitations and future directions for research

The current study had a number of methodological limitations. The retrospective nature of data collection limited us to correlation-based analyses and precluded examination of causal relationships among the variables. As noted above, the study did not include a non-intervention control group, limiting our ability to attribute post-intervention improvements to the rehabilitation interventions. Another limitation, also related to the retrospective nature of the study, involves the lack of information on neurophysiological and neurocognitive measures, particularly at Time 2, which could potentially be associated with the outcome variables. Though the unique data, the large size of the sample, and information on the vocational status of participants in the years prior to beginning intervention do allow us to draw initial conclusions, the relationships between time-since-injury, age at injury onset, and vocational rehabilitation outcomes should be addressed in future prospective, controlled research. To extend and elaborate on the current findings, such research should address specific processes and mechanisms that might explain age-related differences in the relationship between time-since-injury and perceived quality of life following vocational rehabilitation. For example, future studies might address whether employment-related emotional processes experienced by younger survivors, who have often spent less time in the workforce, differ from those of older, more experienced survivors. Additional age-related factors, such as marriage and family status, or social priorities, might also have significant effects on an individual’s capacity and motivation for vocational rehabilitation and on the self-awareness and maturity required to succeed.

### 4.2. Conclusion

Returning to work following brain injury contributes enormously to mental health and perceived life satisfaction (Libeson et al., 2020). Quality of life is reportedly more strongly connected to employment than to any other post-injury variable, though there are many, including severity of cognitive and physical impairment (Ditchman et al., 2022). This highlights the importance of research examining the potential determinants of successful vocational rehabilitation, including the optimal starting point in terms of time from injury and age at intervention onset. The current study shows that among younger participants, there can be value in starting vocational components of rehabilitation later, after they have had the chance to mature and gain relevant experience both independently and through other rehabilitation processes. Meanwhile, among older adults, the results suggest that the effectiveness of vocational rehabilitation can be maximized by starting as early as possible. Most importantly, regardless of age, vocational rehabilitation can be effective even when initiated many years after injury.

## Data availability statement

The data analyzed in this study is subject to the following licenses/restrictions: The dataset is part of a broader dataset recording the clinical outcomes of clients at a neuropsychological rehabilitation center. Requests to access these datasets should be directed to corresponding author.

## Ethics statement

The studies involving human participants were reviewed and approved by National Institute of Neuropsychological Rehabilitation Ethics and Research Committee. The patients/participants provided their written informed consent to participate in this study.

## Author contributions

AB, TS-U, LS, NB-L, SM, and DH contributed to the conception and design of the study. AB and SM wrote the manuscript. TS-S organized the database. ED performed the statistical analysis. All authors contributed to revision and approved the manuscript.

## Conflict of interest

The authors declare that the research was conducted in the absence of any commercial or financial relationships that could be construed as a potential conflict of interest.

## Publisher’s note

All claims expressed in this article are solely those of the authors and do not necessarily represent those of their affiliated organizations, or those of the publisher, the editors and the reviewers. Any product that may be evaluated in this article, or claim that may be made by its manufacturer, is not guaranteed or endorsed by the publisher.
